# HPV seropositivity joints with susceptibility loci identified in GWASs at apoptosis associated genes to increase the risk of Esophageal Squamous Cell Carcinoma (ESCC)

**DOI:** 10.1186/1471-2407-14-501

**Published:** 2014-07-09

**Authors:** Ju Yang, Huanlei Wu, Sheng Wei, Huihua Xiong, Xiangning Fu, Zhaozhen Qi, Qian Jiang, Wen Li, Guangyuan Hu, Xianglin Yuan, Zhongxing Liao

**Affiliations:** 1 Departments of Oncology, Tongji Hospital, Tongji Medical College, Huazhong University of Science and Technology, Wuhan, China; 2 Department of Thoracic Surgery, Tongji Medical College, Huazhong University of Science and Technology, Wuhan, China; 3 Department of Public Health, Tongji Medical College, Huazhong University of Science and Technology, Wuhan, China; 4 Department of Radiation Oncology, Unit 97, The University of Texas MD Anderson Cancer Center, 1515 Holcombe Blvd, Houston, Texas 77030, USA

**Keywords:** Esophageal squamous cell carcinoma, Apoptosis, Genome-wide association study, HPV16, Single nucleotide polymorphism, Smoking, Drinking

## Abstract

**Background:**

We previously showed that human papillomavirus (HPV) serostatus was not an independent risk factor for esophageal squamous cell carcinoma(ESCC) in nonsmokers and nondrinkers; however, HPV increased the risk in smokers.

**Methods:**

Here we investigated possible interactions between HPV16 serostatus and three susceptibility loci identified in GWASs at apoptosis associated genes with regard to risk of ESCC in a case–control study of 313 patients with ESCC and 314 healthy controls. The loci (CHK2 rs738722, C12orf51 rs2074356, and PLCE1 rs2274223) were genotyped, and the presence or absence of HPV16 in serum was measured by ELISA. Multivariable logistic regression was used to evaluate possible interactions of HPV16 serostatus and the three loci on the risk of ESCC.

**Results:**

A significant interaction was found between HPV16 serology and rs2074356 (*P* = 0.005, odds ratio [OR] 1.40, 95% confidence interval [CI] 1.11–1.77) or rs2274223 (*P* < 0.001, OR 1.53, 95% CI 1.23–1.91), but not for rs738722. For rs2074356, risk of ESCC was increased substantially in smokers (*P* < 0.001, OR 8.25, 95% CI 3.84–17.71) and drinkers (OR4.04, *P* = 0.001, 95% CI 1.79–9.10) who carried risk alleles (TT or TC genotype) and were HPV16-seropositive. Similar results were observed for rs2274223 in smokers (*P* < 0.001, OR6.06, 95% CI 2.85–12.88) and drinkers (*P* < 0.001, OR 5.43, 95% CI 2.51–11.76), but not for rs738722.

**Conclusion:**

Consistent with the previous study, loci at rs2074356 and rs2274223 could increase the risk of ESCC, furthermore, there were significant interactions between HPV sero-status and the susceptibility loci on the risk of ESCC. This effect could be modified obviously by smoking and drinking.

## Background

Esophageal cancer is the sixth leading cause of cancer-related death globally. An estimated 482,300 new esophageal cancer cases were diagnosed and 406,800 died of esophageal cancer worldwide in 2008. In high-risk areas such as northern Iran, Central Asian countries, and north central China, esophageal squamous cell carcinoma (SCC) accounts for 90% of esophageal cancers [1]. Poor nutritional status, low intake of fruits and vegetables, drinking hot beverages, and smoking all contribute to the etiology of ESCC [2-4]. In 1982, Syrjanen and colleagues implicated human papillomavirus (HPV) infection in the development of squamous cell papillomas of the esophagus, [5], but the role of HPV infection in esophageal cancer remains controversial.

HPV infection is a risk factor for the development of several types of tumors, particularly HPV16 and HPV18 infection and cervical cancer [6]. HPV16 was reported to be an important infectious factor in the high incidence of esophageal cancer in China [7]. The HPV early protein, E6 can inhibit the activation of apoptosis through binding to p53 and targeting it for degradation, and further cause the malignant transformation [8]. Although HPV infection combined with smoking and drinking could increase the risk of ESCC [9], only a fraction of ESCCs are associated with HPV infection, implying that other genetic factors can modify the association between HPV infection and the risk of ESCC.

There were many newly identified susceptibility loci on the risk of ESCC in the recently published GWAS studies [10-12]. Based on these three studies, significant susceptibility loci in apoptosis-related genes, including loci in 10q23, 22q12 and 12q24, were chosen. PLCE1 (phospholipase C epsilon gene 1) at 10q23 interacts with the proto-oncogene ras and acts on GTPases involved in regulating cell growth, differentiation, apoptosis, and angiogenesis [13,14]. As five susceptibility loci located in PLCE1 at 10q23 had strong pair-wise linkage disequilibrium, we chose the most significant locus rs2274223. This locus was not only a shared locus for these three GWAS studies, but also a shared susceptibility locus for both ESCC and Gastric cancer [10]. CHK2 at 22q12, participates in the Chk2-p53-PUMA signaling pathway, is a major regulator of apoptosis induced by DNA damage in response to double-strand breaks in vivo [15]. As for 22q12, only one susceptibility locus rs738722 located in CHK2 appeared significantly [10]. C12orf51 transcript on 12q24 and has been reported to be in high linkage disequilibrium with SNPs in ALDH2 and ALDH2 was reported to be associated with cardiomyocyte apoptosis post myocardial infarction induced by microRNA-34A, and H₂O₂-induced apoptosis in peripheral blood mononuclear cells [16,17]. As for 12q24, three variants in high linkage disequilibrium conferred their risks to ESCC in a gene-life style interaction manner, with more pronounced risk enhancement seen in tobacco and alcohol users. Among these three variants located in 12q24, only association with rs2074356 remained genome-wide significant after adjusting for the effects of the other two variants, indicating rs2074356 could be an independent susceptibility marker [11]. Therefore, rs2074356 was chosen.

To our knowledge, no studies have been performed to investigate the interactions of the three SNPs CHK2 rs738722, C12orf51 rs2074356, and PLCE1 rs2274223 at apoptosis associated genes and HPV16 serostatus on the risk of ESCC. Therefore, in this study, we analyzed interactions among HPV16 seropositivity and these susceptibility loci on ESCC risk. Because drinking and smoking are risk factors for ESCC in the general population [18], we also investigated the joint effects of smoking or drinking, the selected loci, and HPV16 seropositivity on ESCC risk.

## Methods

### Cases and controls

This study included 313 patients with ESCC and 314 healthy controls, all genetically unrelated Han Chinese. The 313 patients with ESCC were consecutive cases seen at Tongji Hospital between 2009 and 2012 who had histopathologically confirmed primary ESCC. Patients who had primary tumors outside of the esophagus, tumors of unknown origin, or any histopathologic diagnosis other than ESCC were excluded. Healthy controls were volunteers from Tongji Hospital Physical Center who had received a comprehensive health examination and were found to be cancer-free. All of the cases and controls come from Hubei Province. In addition, healthy controls were matched to cases on age and sex.

Eligible participants were interviewed by trained staff using a questionnaire to collect demographic data (age, sex, address) and related exposure information, including tobacco and alcohol use. Participants who smoked at least one cigarette per day for at least 1 year were classified as “ever-smoking” and others were defined as “nonsmoking”. Participants who drank alcoholic beverages at least once a week for more than 1 year were classified as “ever-drinking” and others were classified as “nondrinking”. After the interview, 2-mL peripheral blood samples were collected from each participant and stored at -80°C until genotyping, as described below.

Written informed consent was obtained from each participant before recruitment. This study was approved by the ethics committee of the Tongji Medical College.

### Genotyping

Genomic DNA was isolated from peripheral blood samples by using a FUJI whole blood DNA kit (Fujifilm Corporation, Japan). The three SNPs (rs738722, rs2074356, and rs2274223) were genotyped by a Taqman real-time polymerase chain reaction method using a 7900 HT sequence detector system (Applied Biosystems, Foster City, CA, USA). Probes for these three SNPs were ordered from Applied Biosystems. Allelic discrimination was measured automatically by Sequence Detection Systems 2.3 software (Applied Biosystems). For each SNP, more than 98% of the samples were genotyped successfully. To verify the reproducibility of the Taqman genotyping results, 10% of the samples were randomly selected and retested. The concordance was 99%.

### HPV16 serologic testing

We tested the participants’ serum antibody IgG levels against the HPV16 L1 capsid protein by using an HPV16 L1-capsid antibody ELISA kit (Cusabio, Wuhan, China) according to the manufacturer’s instructions. The cutoff level for HPV16 L1 seropositivity was determined according to the manufacturer’s instructions. We randomly chose 5% of the samples and obtained 100% concordance on the repeat assay.

### Statistical analysis

Distributions of demographic characteristics (age, sex), exposure factors (smoking, drinking, and HPV16 serostatus) and genotypes of the PLCE1 rs2274223, C12orf51 rs2074356 and CHK2 rs738722 variants between cases and controls were examined with chi-square tests. Potential associations of HPV16 and the three susceptibility loci genotypes with the risk of ESCC were assessed by computing odds ratios (ORs) and their 95% confidence intervals (CIs) by using multivariable logistic regression analyses. Associations between HPV16 and ESCC risk were stratified by age, sex, and smoking and drinking status. Trends in the risk of ESCC associated with the three susceptibility loci genotypes were estimated and adjusted for age, sex, smoking and drinking status, and HPV16 serostatus. Tests for linear trends were performed by treating ordered categorical variables as continuous variables in the regression analysis. *P* values for gene*HPV16 interactions were calculated by conducting a 1-degree-of-freedom parameter (SNP*HPV16) in an unconditional logistic regression with age, sex, smoking, and drinking as covariates [19]. The joint effects of HPV16 and the three susceptibility loci genotypes were also estimated and stratified by smoking and drinking. Statistical analyses were performed with SPSS 16.0 (SPSS Inc., Chicago, IL, USA). The Hardy–Weinberg equilibrium for genotype distribution in controls was tested by a goodness-of-fit chi-square test. All tests were 2-sided and *P* values <0.05 were considered to indicate statistically significant differences.

## Results

### Demographic variables and risk factors among study subjects

We initially enrolled 332 people with esophageal cancer as cases in this study, later excluding 19 individuals (2 for unknown histopathology, 14 for not having DNA samples available, and 3 for failure of genotyping), for a total of 313 ESCC cases and 314 healthy controls. Among the cases, mean age was 58.6 ± 8.5 years and 85% were male; among the controls, mean age of controls was 57.7 ± 12.1 years, and 84% were male. (*P* for age 0.247, *P* for sex 0.673), indicating adequate frequency matching on age and sex (Table 1). A T-test for comparison on mean ages between cases and healthy controls was performed (*P* = 0.289). A much higher proportion of cases smoked tobacco than controls (70.6% vs. 40.7%, *P* < 0.001), and more cases drank alcohol than controls (63.3% vs. 50.3%, *P* = 0.001); moreover, more cases were HPV16 seropositivity (54.3%) than controls (43.3%, *P* = 0.006).

**Table 1:** Distribution of demographic variables and risk factors in esophageal squamous cell cancer cases and cancer-free control subjects

**Characteristics**	**Cases (n = 313)**		**Controls (n = 314)**		***P*** **values**	OR (95% CI)*
	**Number**	**(%)**	**Number**	**(%)**		
**Age (years)**					0.247	
**<58**	152	(48.6)	167	(53.2)		1
**≥58**	161	(51.4)	147	(46.8)		1.20 (0.88-1.65)
**Sex**					0.673	
**Female**	47	(15.0)	51	(16.2)		1
**Male**	266	(85.0)	263	(83.8)		1.10 (0.71-1.69)
**Tobacco smoking**					<0.001	
**Never**	92	(29.4)	158	(50.3)		1
**Ever**	221	(70.6)	156	(49.7)		2.43 (1.75-3.38)
**Alcohol drinking**					0.001	
**Never**	115	(36.7)	156	(49.7)		1
**Ever**	198	(63.3)	158	(50.3)		1.70 (1.24-2.34)
**HPV status**						
**-**	143	(45.7)	178	(56.7)	0.006	1
**+**	170	(54.3)	136	(43.3)		1.56 (1.14-2.13)

*P* values for cases vs. controls were calculated with two-sided χ^2^ tests.

*ORs were assessed in logistic regression models.

### Stratified analysis of ESCC risk by HPV16 L1 status

For the group as a whole, risk of ESCC was significantly higher among people who were HPV16-seropositive versus HPV16-seronegative (*P* = 0.001, OR 1.72, 95% CI 1.24–2.39) (Additional file 1: Table S1). Among those who had ever smoked, the risk of ESCC was also significantly higher among HPV16-seropositive than HPV16-seronegative participants (*P* = 0.003, OR 1.91, 95% CI 1.25–2.93). Similar results were also observed among participants older than 58 years (*P* = 0.006, OR 1.98, 95% CI 1.22–3.21), participants who were male (*P* = 0.001, OR 1.85, 95% CI 1.29–2.65), and participants who had ever drank (*P* = 0.002, OR 2.03, 95% CI 1.30–3.17). Among participants who did not smoke or drink, those who were HPV16-seropositive did not have significantly higher risk of ESCC than those who were HPV16-seronegative (*P* = 0.355, OR 1.35, 95% CI 0.72–2.52).

Among nondrinking and HPV16-seropositive subjects, those who had ever smoked were at significantly higher risk of ESCC than were non-smokers (*P* = 0.034, OR 2.26, 95% CI 1.06–4.79) (Additional file 1: Table S2). However, among non-smoking and HPV16-seropositive subjects, those who drank were not at significantly higher risk. Risk of ESCC was substantially higher among participants who smoked, drank, and were HPV-seropositive (*P* < 0.001, OR 5.03, 95% CI 2.66–9.56) compared with those who did not smoke, did not drink, and were HPV-seropositive.

### Genotype distribution of susceptibility loci and association with ESCC risk

Genotype frequencies of the three selected SNPs in cases and controls are summarized in Table 2. The observed genotype frequencies among controls were in agreement with the Hardy-Weinberg equilibrium (*P* = 0.689 for rs738722, *P* = 0.433 for rs2074356, and *P* = 0.609 for rs2274223). For rs738722, no significant difference was found in the distribution of three genotypes between cases and controls (*P* = 0.361). The TT and CT genotypes were more frequent among cases than controls (TT 7.0% vs. 5.7%; CT 39.0% vs. 34.7%), but ESCC risk was not increased among those with the TT genotype (OR 1.34, 95% CI 0.68–2.66) or those with the CT genotype (OR 1.22, 95% CI 0.87–1.73). For rs2074356, compared with the GG genotype, significantly increased risk of ESCC was associated with the AG genotype (OR 1.61, 95% CI 1.12–2.30) and the combined AA/AG genotypes (OR 1.52, 95% CI 1.07–2.16). For rs2274223, compared with the AA genotype, significantly increased risk of ESCC was associated with the GG genotype (OR 2.86, 95% CI 1.22–6.71), the AG genotype (OR 1.70, 95% CI 1.20–2.41) and the combined GG/AG genotypes (OR 1.75, 95% CI 1.25–2.46). For rs2074356 and rs2274223, the risk of ESCC may have increased with increasing numbers of variant alleles (*P*_trend_ = 0.019 for rs2074356 and *P*_trend_ = 0.001 for rs2274223) (Table 2).

**Table 2:** Association of susceptibility loci identified in previous GWAS with ESCC risk in cases and controls

**Susceptibility Loci**	**Cases (n = 313)**		**Controls (n = 314)**		***P*** values*	**Adjusted OR (95% CI)**^**†**^
	**No.**	**%**	**No.**	**%**		
**rs738722**^**α**^					0.361	
**CC (Ref.)**	169	54.0	187	59.6		1.00
**CT**	122	39.0	109	34.7		1.22 (0.87-1.73)
**TT**	22	7.0	18	5.7		1.34 (0.68-2.66)
**Trend test**						*P* = 0.242
**TT + CT**	144	46.0	127	40.4		1.22 (0.88-1.69)
**rs2074356**^**β**^					0.011	
**CC (Ref.)**	196	62.6	227	72.3		1.00
**TT**	2	0.6	5	1.6		0.61 (0.12-3.22)
**TC**	115	36.7	82	26.1		1.61 (1.12-2.30)
**Trend test**						*P* = 0.019
**TT + TC**	117	37.3	87	27.7		1.52 (1.07-2.16)
**rs2274223**^**γ**^					0.006	
**AA (Ref.)**	172	55.0	209	66.6		1.00
**AG**	122	39.0	96	30.6		1.70 (1.20-2.41)
**GG**	19	6.1	9	2.9		2.86 (1.22-6.71)
**Trend test**						*P* = 0.001
**GG + AG**	141	45.1	105	33.5		1.75 (1.25-2.46)

*Genotype distribution of rs738722, rs2074356 and rs2274223 was assessed by Chi-square test.

^†^ORs were adjusted for age, sex, smoking, drinking and HPV16 status.

^α^The observed genotype frequencies of rs738722 among controls were in agreement with Hardy-Weinberg equilibrium (*P* = 0.689).

^β^The observed genotype frequencies of rs2074356 among controls were in agreement with Hardy-Weinberg equilibrium (*P* = 0.433).

^γ^The observed genotype frequencies of rs2274223 among controls were in agreement with Hardy-Weinberg equilibrium (*P* = 0.609).

### Interactions of C12orf51 rs2074356 and PLCE1 rs2274223 on the risk of ESCC

As indicated in Table 3, patients with rs2074356 CT/TT genotypes and rs2274223 GG/AG genotypes simultaneously had increased risk of ESCC, compared to patients with rs2074356 CC and rs2274223 AA genotypes (OR 3.31, 95% CI 1.87–5.83). There was an interaction between rs2074356 and rs2274223 on the risk of ESCC (OR 1.26, 95% CI 1.09–1.45).

**Table 3:** Combined effect of rs2074356 and rs2274223 on the risk of ESCC

**Rs2074356**	**Rs2274223**	**Cases (n = 313)**		**Controls (n = 314)**		**Adjusted** ***P*** **value**	Adjusted OR (95% CI)*
		**No.**	**%**	**No**	**%**		
**CC**	**AA**	109	34.8	146	46.5		1
**CC**	**GG/AG**	87	27.8	81	25.8	0.024	1.60 (1.06-2.42)
**CT/TT**	**AA**	63	20.1	63	20.1	0.211	1.33 (0.85-2.08)
**CT/TT**	**GG/AG**	54	17.3	24	7.6	<0.001	3.31 (1.87-5.83)
Rs2074356*rs2274223						0.002	1.26 (1.09-1.45)

*ORs were adjusted for age, sex, smoking and drinking in logistic regression models.

### Interactions of HPV16 seropositivity and SNPs at susceptibility loci on the risk of ESCC

As shown in Table 4, compared to HPV16-seronegative subjects with the rs738722 CC genotype, the risk of ESCC increased among HPV16-seropositive participants with the CC genotype (*P* = 0.002, OR 2.00, 95% CI 1.29–3.10) and among HPV16-seropositive subjects with TT or CT genotype (*P* = 0.003, OR 2.09, 95% CI 1.29–3.38). However, the interaction between rs738722 genotypes and HPV16 serology for ESCC risk was not significant (*P* = 0.068). Compared to HPV16-seronegative participants with the rs2074356 GG genotype, ESCC risk increased among HPV16-seropositive subjects with the GG genotype (*P* = 0.005, OR 1.78, 95% CI 1.19–2.66) and among HPV16-seropositive participants with the AA or AG genotype (*P* < 0.001, OR 2.66, 95% CI 1.59–4.46). Here the interaction between rs2074356 genotypes and HPV16 serology for ESCC risk was significant (*P* = 0.005, OR 1.40, 95% CI 1.11–1.77). Compared with HPV16-seronegative participants with the rs2274223 AA genotype, the risk of ESCC was elevated among HPV16-seropositive subjects with the AA genotype (*P* = 0.042, OR 1.55, 95% CI 1.02–2.37) and HPV16-seropositive participants with the GG or AG genotype (*P* < 0.001, OR 3.17, 95% CI 1.94–5.17). The interaction between rs2274223 genotypes and HPV16 serology for ESCC risk was also significant (*P* < 0.001, OR 1.53, 95% CI, 1.23–1.91).

**Table 4:** Joint effects and interactions of HPV16 L1 seropositivity and genotypes at susceptibility loci on the risk of esophageal squamous cell carcinoma in cases and controls

**HPV16 status**	**Susceptibility Loci**	**Cases (n = 313)**		**Controls (n = 314)**		**Adjusted** ***P*** **values**	Adjusted OR (95% CI)^*^
		**No.**	**%**	**No.**	**%**		
	**Rrs738722**						
**Negative**	**CC (Ref.)**	70	22.4	106	33.8		1.00
**Negative**	**TT + CT**	73	23.3	72	22.9	0.112	1.45 (0.92-2.30)
**Positive**	**CC**	99	31.6	81	25.8	0.002	2.00 (1.29-3.10)
**Positive**	**TT + CT**	71	22.7	55	17.5	0.003	2.09 (1.29-3.38)
	G*HPV16					0.068	1.223 (0.99-1.52)
	**rsrs2074356**						
**Negative**	**CC (Ref.)**	87	27.8	128	40.8		1.00
**Negative**	**TT + TC**	56	17.9	50	15.9	0.054	1.61 (0.99-2.62)
**Positive**	**CC**	109	33.9	99	31.5	0.005	1.78 (1.19-2.66)
**Positive**	**TT + TC**	61	19.5	37	11.8	<0.001	2.66 (1.59-4.46)
	G*HPV16					0.005	1.40 (1.11-1.77)
	**rsrs2274223**						
**Negative**	**AA (Ref.)**	82	26.2	118	37.6		1.00
**Negative**	**GG + AG**	61	19.5	60	19.1	0.055	1.59 (0.99-2.55)
**Positive**	**AA**	90	28.8	91	29.0	0.042	1.55 (1.02-2.37)
**Positive**	**GG + AG**	80	25.6	45	14.3	<0.001	3.17 (1.94-5.17)
	GG*HPV16					<0.001	1.53 (1.23-1.91)

*ORs were adjusted for age, sex, smoking and drinking in logistic regression models.

*P* values for gene*HPV16 interaction were calculated by conducting a 1-degree-of-freedom parameter (SNP*HPV16) as implemented in unconditional logistic regression with age, sex, smoking and drinking as covariates (22).

### Stratified analysis of the joint effect of HPV16 seropositivity and SNPs at susceptibility loci on ESCC risk by smoking or drinking status

**Figure 1:**
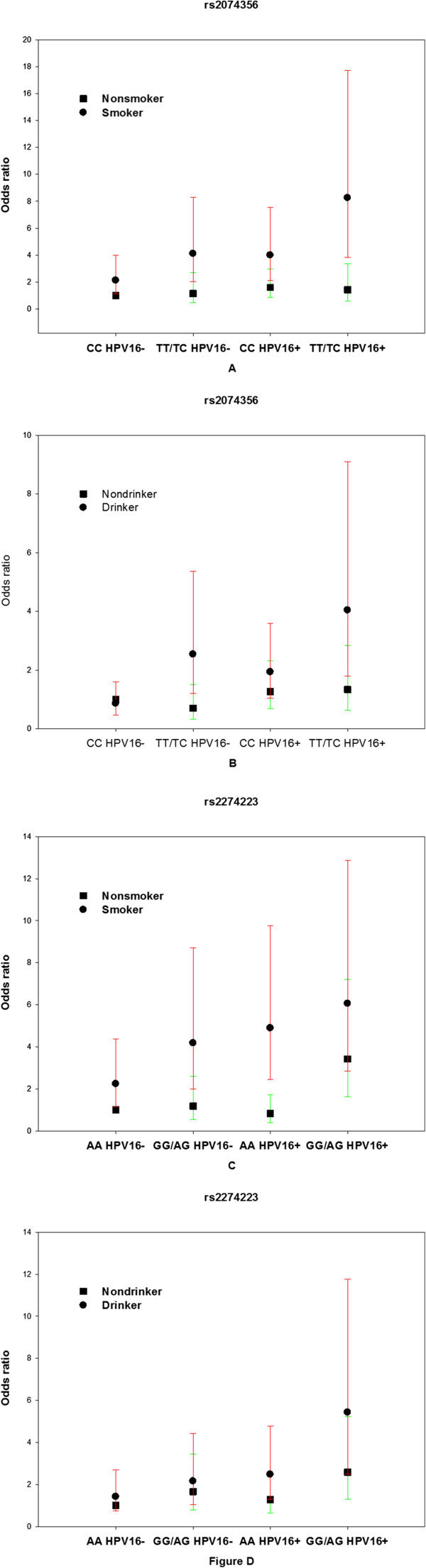
**Odds ratios for esophageal squamous cell carcinoma (ESCC) in smokers/drinkers and nonsmokers/nondrinkers with different rs2074356/rs2274223 genotypes and HPV16 serology. A,** Odds ratios for ESCC in smokers and nonsmokers with different rs2074356 and HPV16 serology. Non-smoker, CC and HPV16- was reference for **A**. **B**, Odds ratios for ESCC in drinkers and nondrinkers with different rs2074356 and HPV16 serology. Non-drinker, CC and HPV16- was reference for **B**. **C**, Odds ratios for ESCC in smokers and nonsmokers with different rs2274223 and HPV16 serology. Non-smoker, AA and HPV16- was reference for **C**. **D**, Odds ratios for ESCC in drinkers and nondrinkers with different rs2274223and HPV16 serology. Non-drinker, AA and HPV16- was reference for Figure **D**. The vertical bars represent the 95% confidence intervals.

As shown above, HPV16 seropositivity synergized with different rs2074356 or rs2274223 genotypes to increase the risk of ESCC, but this synergy was not observed for rs738722. In the general population, smoking and drinking are known risk factors for ESCC [18,20], so we further stratified the joint effect of HPV16 serology and the three selected SNPs by smoking or drinking. For rs2074356, the OR in HPV16-seropositive smokers carrying risk alleles (TT or TC genotype) was more than six times higher than that in HPV16-seronegative smokers carrying the non-risk alleles (CC genotype), and was approximately seven times higher than for HPV16-seropositive nonsmokers carrying risk alleles (TT or TC genotype) (Figure 1A). Similar results were observed for the analysis of the joint effects of drinking, rs2074356, and HPV16 serology (Figure 1B). The effect sizes of rs2074356 and HPV16 serology in nonsmokers or nondrinkers were not large (Figures 1A and B). For rs2274223, the OR in HPV16-seropositive smokers carrying risk alleles (GG or AG genotype) was nearly four times higher than for HPV16-seronegative smokers carrying non-risk alleles (AA genotype), and was approximately three times higher than for HPV16-seropositive nonsmokers carrying risk alleles (GG or AG genotype) (Figure 1C). Similar results were seen for the analysis of the joint effects of drinking, rs2274223, and HPV16 serology (Figure 1D). For rs738722, risk of ESCC was not increased among HPV16-seropositive smokers or drinkers with GG/AG alleles compared with HPV16-seropositive nonsmokers or nondrinkers with GG/AG alleles (data not shown).

## Discussion

We found that genotypes at rs2274223 and rs2074356 contributed to the risk of ESCC independently of HPV16 seropositivity, as had been found in previous genome-wide association studies; however, we found that rs738722 did not contribute to the risk of ESCC. Although HPV16 serostatus was not an independent risk factor for ESCC in nonsmokers and nondrinkers, the interaction of HPV16 serostatus and rs2074356 or rs2274223 significantly increased the risk of ESCC in these subjects. In addition, smoking and drinking synergized with the joint effect of HPV16 serostatus and rs2074356 or rs2274223 to substantially increase ESCC risk. To our knowledge, our study is the first to analyze interactions of HPV16 seropositivity and genotypes at apoptosis associated genes for the risk of ESCC.

Our multivariable logistic regression models showed no association between rs738722 and the risk of ESCC after adjusting for age, sex, smoking, drinking, and HPV16 status. This is consistent with findings from a previous genome-wide association study that the apparent association between rs738722 and ESCC risk was not statistically significant in the second phase (*P*_trend_ = 0.14, OR 1.20, 95% CI 0.94–1.53) [10]. Another study also reported that variant alleles of CHEK2 rs738722 were not associated with risk of ESCC [21]. Further study is needed to investigate the association between rs738722 and ESCC risk.

Findings from studies of HPV16 and risk of ESCC have been inconsistent [22-24]. Testing methods, demographic and ethnic factors, and exposure to other ESCC risks may contribute to the inconsistencies among the results. Similar to the methods used in our study, serological markers of HPV have been used in other studies to investigate potential associations between HPV serostatus and ESCC risk [25-28]. The effect of HPV16 seropositivity on ESCC risk in our current study is consistent with our previous study [29]. The current study included a larger number of cases (313 vs. 225) and a better method of identifying HPV seropositivity via identification of antibodies against HPV16 L1 rather than merely high-risk HPV.

Similar studies have been done to investigate joint effects of genetic polymorphisms and HPV infection on risk of ESCC. HLA-DRB1*1501 and HLA-DQB1*0301 was reported to influence HPV-encoded epitopes and affect the risk of ESCC among Kazakhs in XinJiang, China [30]. Our another previous study demonstrated that HPV16 seropositivity synergized with p53 Arg/Arg or Arg/Pro and increased ESCC risk, especially in smokers or drinkers [31]. However, until now no studies have investigated the joint effect of HPV16 seropositivity, susceptibility loci, and the effects of smoking and drinking on risk of ESCC.

Results of this study were also biologically reasonable. After HPV infection, the E6 protein can bind to p53 through E6AP and prevent p53 from inducing apoptosis through targeting it for degradation via the ubiquitin–proteasome pathway [32]. Risk alleles at apoptosis genes may contribute to the inhibition of apoptosis and this could explain our results why HPV could synergize with susceptibility loci identified in GWASs at apoptosis associated genes to increase the risk of ESCC.

Our study did have some limitations, including the fact that all of the individuals enrolled in our study were ethnic Han Chinese and all of them were from Hubei Province. Our study is retrospective and hospital based. All of the controls were from Hubei Province, China, received a comprehensive examination of health and found to be cancer free. The majority of the controls come from city and there are more rural residents in cases, therefore, there may be differences of the lifestyle, social economic status (SES), education level, food habits and so on between controls and cases. However, in this study, information of these factors is not accessible. Lack of the information of these factors may resulted in selection bias and limited representativeness of samples. In our further research, lifestyle, SES, education level, and food habits will be evaluated for the new participants to reduce the selection bias and limited representativeness of the samples. Our study still needs to be further validated in other parts of China or other ethnics.

## Conclusions

In summary, we found that SNPs at two susceptibility loci identified in previous genome-wide association studies (rs2274223 and rs2074356) increased the risk of ESCC independent of HPV16 seropositivity, but a third SNP (rs738722) did not. HPV16 serostatus was not an independent risk factor in nonsmokers and nondrinkers. However, interactions between HPV16 seropositivity and risk alleles of rs2074356 or rs2274223 increased the risk of ESCC substantially, especially in smokers or drinkers.

## Competing interests

The authors declare that they have no competing interests.

## Authors’ contributions

JY recruited patients, collected the data, performed the experiment and drafted the manuscript. HW, SW, HX, XF, ZQ, QJ, GH, WLparticipated in patients’recruitment, study materials collection and experiment design. XY and ZL participated in the design of the study, interpretation of data and revision of the manuscript. All authors read and approved the final manuscript.

## Pre-publication history

The pre-publication history for this paper can be accessed here:

http://www.biomedcentral.com/1471-2407/14/501/prepub

## Supplementary Material

Additional file 1: Table S1 Associations between age, sex, smoking, drinking, and risk of ESCC stratified by HPV16 infection. **Table S2.** Stratification of risk of ESCC in HPV seropositive group by smoking and alcohol. **Table S3.** Odds ratios (ORs) for ESCC in smokers and nonsmokers with different rs738722 and HPV16 serology. Non-smoker, CC and HPV16- was reference for Table S3. **Table S4.** ORs for ESCC in drinkers and nondrinkers with different rs738722 and HPV16 serology. **Table S5.** ORs for ESCC in smokers and nonsmokers with different rs2074356 and HPV16 serology. **Table S6.** ORs for ESCC in drinkers and nondrinkers with different rs2074356 and HPV16 serology. **Table S7.** ORs for ESCC in smokers and nonsmokers with different rs2274223 and HPV16 serology. **Table S8.** ORs for ESCC in drinkers and nondrinkers with different rs2274223 and HPV16 serology. **Table S9.** Combined effect of HPV, rs2074356 and rs2274223 on the risk of ESCC. **Table S10.** The association between smoking or drinking and HPV sero status in controls. **Table S11.** The association between smoking or drinking and HPV sero status in patients with ESCC. **Table S12.** The association between age, sex, drinking, smoking and rs738722 in controls. **Table S13.** The association between age, sex, drinking, smoking and rs738722 in patients with ESCC. **Table S14.** The association between age, sex, drinking, smoking and rs2074356 in controls. **Table S15.** The association between age, sex, drinking, smoking and rs2074356 in patients with ESCC. **Table S16.** The association between age, sex, drinking, smoking and rs2274223 in controls. **Table S17.** The association between age, sex, drinking, smoking and rs2274223 in patients with ESCC. **Table S18.** Interactions between snps and age, sex, drinking and smoking on the risk of ESCC. **Table S19.** Multivariate analysis of age, sex, smoking, drinking, HPV sero status, SNPs and the risk of ESCC. Click here for file
