# Supplementary material for: HPV seropositivity joints with susceptibility loci identified in GWASs at apoptosis associated genes to increase the risk of Esophageal Squamous Cell Carcinoma (ESCC)
Source: BMC Cancer. 2014 Jul 9;14:501. doi: 10.1186/1471-2407-14-501 (PMC4227071; doi:10.1186/1471-2407-14-501)
Supplement: Additional file 1: Table S1 — Associations between age, sex, smoking, drinking, and risk of ESCC stratified by HPV16 infection. Table S2. Stratification of risk of ESCC in HPV seropositive group by smoking and alcohol. Table S3. Odds ratios (ORs) for ESCC in smokers and nonsmokers with different rs738722 and HPV16 serology. Non-smoker, CC and HPV16- was reference for Table S3. Table S4. ORs for ESCC in drinkers and nondrinkers with different rs738722 and HPV16 serology. Table S5. ORs for ESCC in smokers and nonsmokers with different rs2074356 and HPV16 serology. Table S6. ORs for ESCC in drinkers and nondrinkers with different rs2074356 and HPV16 serology. Table S7. ORs for ESCC in smokers and nonsmokers with different rs2274223 and HPV16 serology. Table S8. ORs for ESCC in drinkers and nondrinkers with different rs2274223 and HPV16 serology. Table S9. Combined effect of HPV, rs2074356 and rs2274223 on the risk of ESCC. Table S10. The association between smoking or drinking and HPV sero status in controls. Table S11. The association between smoking or drinking and HPV sero status in patients with ESCC. Table S12. The association between age, sex, drinking, smoking and rs738722 in controls. Table S13. The association between age, sex, drinking, smoking and rs738722 in patients with ESCC. Table S14. The association between age, sex, drinking, smoking and rs2074356 in controls. Table S15. The association between age, sex, drinking, smoking and rs2074356 in patients with ESCC. Table S16. The association between age, sex, drinking, smoking and rs2274223 in controls. Table S17. The association between age, sex, drinking, smoking and rs2274223 in patients with ESCC. Table S18. Interactions between snps and age, sex, drinking and smoking on the risk of ESCC. Table S19. Multivariate analysis of age, sex, smoking, drinking, HPV sero status, SNPs and the risk of ESCC. [file 1471-2407-14-501-S1.docx]

**Table S1.** Associations between age, sex, smoking, drinking, and the risk of esophageal squamous cell carcinoma stratified by HPV16 infection

| **Characteristics** | **HPV16 status** | **Cases (n=313)** | | **Controls (n=314)** | | **Adjusted *P* value** | **Adjusted OR (95% CI)^*^** |
| --- | --- | --- | --- | --- | --- | --- | --- |
|  |  | No. | (%) | No. | (%) |  |  |
| **All** | - | 143 | 45.7 | 178 | 56.7 | 0.001 | 1 |
|  | + | 170 | 54.3 | 136 | 43.3 |  | 1.72 (1.24-2.39) |
| **Age** |  |  |  |  |  |  |  |
| **<58** | - | 70 | 22.4 | 92 | 29.3 | 0.071 | 1 |
|  | + | 82 | 26.2 | 75 | 23.9 |  | 1.52 (0.97-2.40) |
| **≥58** | - | 73 | 23.3 | 86 | 27.4 | 0.006 | 1 |
|  | + | 88 | 28.1 | 61 | 19.4 |  | 1.98 (1.22-3.21) |
| **Sex** |  |  |  |  |  |  |  |
| **Female** | - | 26 | 8.3 | 30 | 9.6 | 0.864 | 1 |
|  | + | 21 | 6.7 | 21 | 6.7 |  | 1.08 (0.46-2.50) |
| **Male** | - | 117 | 37.4 | 148 | 47.1 | 0.001 | 1 |
|  | + | 149 | 47.6 | 115 | 36.6 |  | 1.85 (1.29-2.65) |
| **Smoking** |  |  |  |  |  |  |  |
| **Ever** | - | 101 | 32.3 | 95 | 30.3 | 0.003 | 1 |
|  | + | 120 | 38.3 | 61 | 19.4 |  | 1.91 (1.25-2.93) |
| **Never** | - | 42 | 13.4 | 83 | 26.4 | 0.183 | 1 |
|  | + | 50 | 16.0 | 75 | 23.9 |  | 1.43 (0.85-2.43) |
| **Drinking** |  |  |  |  |  |  |  |
| **Ever** | - | 90 | 28.8 | 95 | 30.3 | 0.002 | 1 |
|  | + | 108 | 34.5 | 63 | 20.1 |  | 2.03 (1.30-3.17) |
| **Never** | - | 53 | 16.9 | 83 | 26.4 | 0.165 | 1 |
|  | + | 62 | 19.8 | 73 | 23.2 |  | 1.42 (0.87-2.33) |
| **Non-smoking and non-drinking** | - | 33 |  | 56 |  | 0.355 | 1 |
|  | + | 35 |  | 50 |  |  | 1.35 (0.72-2.52) |

HPV-16, human papillomavirus type 16; OR, odds ratio; CI, confidence interval; -, seronegative; +, seropositive.

*OR was adjusted for age, sex, smoking and drinking using logistic regression.

**Table S2.** Stratification of risk of ESCC in HPV seropositive group by smoking and alcohol.

|  | **Smoking** | **Drinking** | **Cases (n=313)** | | **Controls (n=314)** | | **Adjusted *P* value** | **Adjusted OR (95% CI)** |
| --- | --- | --- | --- | --- | --- | --- | --- | --- |
|  |  |  | No. | % | No. | % |  |  |
| **HPV+** | - | - | 35 | 11.2 | 50 | 15.9 |  | 1 |
| **HPV+** | + | - | 27 | 8.6 | 23 | 7.3 | 0.034 | 2.26(1.06-4.79) |
| **HPV+** | - | + | 15 | 4.8 | 25 | 8.0 | 0.626 | 1.23(0.54-2.78) |
| **HPV+** | + | + | 93 | 29.7 | 38 | 12.1 | <0.001 | 5.03(2.66-9.56) |

- smoking/drinking: never smoking/drinking; + smoking/drinking: ever smoking/drinking

*ORs were adjusted for age and sex in logistic regression models.

**Table S3**, Odds ratios for ESCC in smokers and nonsmokers with different rs738722 and HPV16 serology. Non-smoker, CC and HPV16- was reference for Table S3.

| Smoking | HPV | Rs738722 | Cases(n=313) | | Controls(n=314) | | Adjusted *P* value | Adjusted OR(95%CI) |
| --- | --- | --- | --- | --- | --- | --- | --- | --- |
|  |  |  | No. | % | No | % |  |  |
| - | - | CC | 21 | 6.7 | 52 | 16.6 |  | 1 |
| - | - | CT/TT | 21 | 6.7 | 31 | 9.9 | 0.246 | 1.57(0.73-3.38) |
| - | + | CC | 27 | 8.6 | 49 | 15.6 | 0.329 | 1.42(0.70-2.87) |
| - | + | CT/TT | 23 | 7.3 | 26 | 8.3 | 0.029 | 2.35(1.09-5.08) |
| + | - | CC | 50 | 16.0 | 54 | 17.2 |  | 1 |
| + | - | CT/TT | 51 | 16.3 | 41 | 13.1 | 0.300 | 1.36(0.76-2.41) |
| + | + | CC | 71 | 22.7 | 32 | 10.2 | 0.003 | 2.38(1.33-4.24) |
| + | + | CT/TT | 49 | 15.7 | 29 | 9.2 | <0.029 | 2.00(1.07-3.70) |

**Table S4**, Odds ratios for ESCC in drinkers and nondrinkers with different rs738722 and HPV16 serology. Non-drinker, CC and HPV16- was reference for Table S4.

| Drinking | HPV | Rs738722 | Cases(n=313) | | Controls(n=314) | | Adjusted *P* value | Adjusted OR(95%CI) |
| --- | --- | --- | --- | --- | --- | --- | --- | --- |
|  |  |  | No. | % | No | % |  |  |
| - | - | CC | 28 | 8.9 | 48 | 15.3 |  | 1 |
| - | - | CT/TT | 25 | 8.0 | 35 | 11.1 | 0.727 | 1.13(0.56-2.29) |
| - | + | CC | 33 | 10.5 | 44 | 14.0 | 0.326 | 1.39(0.72-2.70) |
| - | + | CT/TT | 29 | 9.3 | 29 | 9.2 | 0.165 | 1.65(0.81-3.36) |
| + | - | CC | 43 | 13.7 | 58 | 18.5 |  | 1 |
| + | - | CT/TT | 47 | 15.0 | 37 | 11.8 | 0.099 | 1.67(0.91-3.06) |
| + | + | CC | 65 | 20.8 | 37 | 11.8 | 0.003 | 2.48(1.37-4.47) |
| + | + | CT/TT | 43 | 13.7 | 26 | 8.3 | 0.004 | 2.69(1.38-5.23) |

**Table S5**, Odds ratios for ESCC in smokers and nonsmokers with different rs2074356 and HPV16 serology. Non-drinker, CC and HPV16- was reference for Table S5.

| Smoking | HPV | Rs2074356 | Cases(n=313) | | Controls(n=314) | | Adjusted *P* value | Adjusted OR(95%CI) |
| --- | --- | --- | --- | --- | --- | --- | --- | --- |
|  |  |  | No. | % | No | % |  |  |
| - | - | CC | 30 | 9.6 | 62 | 19.7 |  | 1 |
| - | - | CT/TT | 12 | 3.8 | 21 | 6.7 | 0.783 | 1.13(0.48-2.62) |
| - | + | CC | 38 | 12.1 | 54 | 17.2 | 0.165 | 1.54(0.84-2.85) |
| - | + | CT/TT | 12 | 3.8 | 21 | 6.7 | 0.544 | 1.30(0.56-3.05) |
| + | - | CC | 57 | 18.2 | 66 | 21.0 |  | 1 |
| + | - | CT/TT | 44 | 14.1 | 29 | 9.2 | 0.015 | 2.13(1.57-3.92) |
| + | + | CC | 71 | 22.7 | 45 | 14.3 | 0.014 | 1.95(1.14-3.32) |
| + | + | CT/TT | 49 | 15.7 | 16 | 5.1 | <0.001 | 4.33(2.17-8.65) |

**Table S6**, Odds ratios for ESCC in drinkers and nondrinkers with different rs2074356 and HPV16 serology. Non-drinker, CC and HPV16- was reference for Table S6.

| Drinking | HPV | Rs2074356 | Cases(n=313) | | Controls(n=314) | | Adjusted *P* value | Adjusted OR(95%CI) |
| --- | --- | --- | --- | --- | --- | --- | --- | --- |
|  |  |  | No. | % | No | % |  |  |
| - | - | CC | 37 | 11.8 | 53 | 16.9 |  | 1 |
| - | - | CT/TT | 16 | 5.1 | 30 | 9.6 | 0.405 | 0.73 (0.34-1.54) |
| - | + | CC | 41 | 13.1 | 49 | 15.6 | 0.477 | 1.24 (0.68-2.27) |
| - | + | CT/TT | 21 | 6.7 | 24 | 7.6 | 0.438 | 1.34(0.64-2.83) |
| + | - | CC | 50 | 16.0 | 75 | 23.9 |  | 1 |
| + | - | CT/TT | 40 | 12.8 | 20 | 6.4 | 0.001 | 3.07(1.57-6.02) |
| + | + | CC | 68 | 21.7 | 50 | 15.9 | 0.002 | 2.36(1.38-4.06) |
| + | + | CT/TT | 40 | 12.8 | 13 | 4.1 | <0.001 | 4.94(2.33-10.49) |

**Table S7**, Odds ratios for ESCC in smokers and nonsmokers with different rs2274223 and HPV16 serology. Non-drinker, CC and HPV16- was reference for Table S7.

| Smoking | HPV | Rs2274223 | Cases(n=313) | | Controls(n=314) | | Adjusted *P* value | Adjusted OR(95%CI) |
| --- | --- | --- | --- | --- | --- | --- | --- | --- |
|  |  |  | No. | % | No | % |  |  |
| - | - | AA | 26 | 8.3 | 52 | 16.6 |  | 1 |
| - | - | GG/AG | 16 | 5.1 | 31 | 9.9 | 0.794 | 1.11(0.51-2.42) |
| - | + | AA | 18 | 5.8 | 52 | 16.6 | 0.467 | 0.76(0.37-1.58) |
| - | + | GG/AG | 32 | 10.2 | 23 | 7.3 | 0.002 | 3.18(1.52-6.64) |
| + | - | AA | 56 | 17.9 | 66 | 21.0 |  | 1 |
| + | - | GG/AG | 45 | 14.4 | 29 | 9.2 | 0.036 | 1.90(1.04-3.47) |
| + | + | AA | 72 | 23.0 | 39 | 12.4 | 0.004 | 2.23 (1.30-3.83) |
| + | + | GG/AG | 48 | 15.3 | 22 | 7.0 | 0.002 | 2.82(1.49-5.36) |

**Table S8**, Odds ratios for ESCC in drinkers and nondrinkers with different rs2274223 and HPV16 serology. Non-drinker, CC and HPV16- was reference for Table S8.

| Drinking | HPV | Rs2274223 | Cases(n=313) | | Controls(n=314) | | Adjusted *P* value | Adjusted OR(95%CI) |
| --- | --- | --- | --- | --- | --- | --- | --- | --- |
|  |  |  | No. | % | No | % |  |  |
| - | - | AA | 29 | 9.3 | 54 | 17.2 |  | 1 |
| - | - | GG/AG | 24 | 7.7 | 29 | 9.2 | 0.175 | 1.64(0.80-3.37) |
| - | + | AA | 27 | 8.6 | 45 | 14.3 | 0.536 | 1.24(0.63-2.41) |
| - | + | GG/AG | 35 | 10.8 | 28 | 8.9 | 0.008 | 2.55 (1.28-5.10) |
| + | - | AA | 53 | 16.9 | 64 | 20.4 |  | 1 |
| + | - | GG/AG | 37 | 11.8 | 31 | 9.9 | 0.157 | 1.58(0.84-2.95) |
| + | + | AA | 63 | 19.5 | 46 | 14.6 | 0.036 | 1.81(1.04-3.14) |
| + | + | GG/AG | 45 | 14.4 | 17 | 5.4 | <0.001 | 4.25(2.07-8.74) |

**Table S9** Combined effect of HPV, rs2074356 and rs2274223 on the risk of ESCC

| Rs2074356 | Rs2274223 | HPV | Cases(n=313) | | Controls(n=314) | | Adjusted *P* value | Adjusted OR(95%CI) |
| --- | --- | --- | --- | --- | --- | --- | --- | --- |
|  |  |  | No. | % | No | % |  |  |
| CC | AA | - | 49 | 15.6 | 84 | 26.8 |  | 1 |
| CC | GG/AG | - | 38 | 12.1 | 44 | 14.1 | 0.086 | 1.67(0.93-2.99) |
| CT/TT | AA | - | 34 | 10.8 | 33 | 10.5 | 0.096 | 1.69(0.91-3.13) |
| CT/TT | GG/AG | - | 23 | 7.3 | 16 | 5.1 | 0.015 | 2.57(1.20-5.50) |
| CC | AA | + | 60 | 19.1 | 62 | 19.8 | 0.022 | 1.84(1.09-3.10) |
| CC | GG/AG | + | 49 | 15.6 | 37 | 11.8 | 0.001 | 2.80(1.56-5.02) |
| CT/TT | AA | + | 30 | 9.6 | 29 | 9.3 | 0.049 | 1.92(1.00-3.69) |
| CT/TT | GG/AG | + | 31 | 9.8 | 8 | 2.6 | <0.001 | 8.77(3.56-21.56) |

**Table S10** The association between smoking or drinking and HPV sero status in cancer free controls

|  | Controls(n=314) | | Adjusted *P* value | Adjusted OR(95%CI) |
| --- | --- | --- | --- | --- |
|  | HPV- | HPV+ | |  |
| Never smoking | 83 | 75 | | 1 |
| Ever smoking | 95 | 61 | 0.116 | 0.67(0.40-1.11) |
| Never drinking | 83 | 73 |  | 1 |
| Ever drinking | 95 | 63 | 0.203 | 0.72(0.43-1.20) |

**Table S11** The association between smoking or drinking and HPV sero status in patients with ESCC

|  | Cases(n=313) | | Adjusted *P* value | Adjusted OR(95%CI) |
| --- | --- | --- | --- | --- |
|  | HPV- | HPV+ |  |  |
| Never smoking | 42 | 50 |  | 1 |
| Ever smoking | 101 | 120 | 0.425 | 0.77(0.41-1.45) |
| Never drinking | 53 | 62 |  | 1 |
| Ever drinking | 90 | 108 | 0.693 | 0.89(0.51-1.57) |

Adjusted by age sex

**Table S12** The association between age, sex, drinking, smoking and rs738722 in cancer free controls

|  | Controls(n=314) | | Adjusted *P* value | Adjusted OR(95%CI) |
| --- | --- | --- | --- | --- |
|  | CC | CT/TT |  |  |
| Never smoking | 101 | 57 |  | 1 |
| Ever smoking | 86 | 70 | 0.062 | 1.65(0.98-2.79) |
| Never drinking | 92 | 64 |  | 1 |
| Ever drinking | 95 | 63 | 0.421 | 0.81(0.48-1.36) |
| <58 | 93 | 74 |  | 1 |
| ≥58 | 94 | 53 | 0.124 | 0.69(0.43-1.11) |
| Female | 30 | 21 |  | 1 |
| Male | 157 | 106 | 0.402 | 0.73(0.36-1.51) |

**Table S13** The association between age, sex, drinking, smoking and rs738722 in patients with ESCC

|  | Cases(n=313) | | Adjusted *P* value | Adjusted OR(95%CI) |
| --- | --- | --- | --- | --- |
|  | CC | CT/TT |  |  |
| Never smoking | 48 | 44 |  | 1 |
| Ever smoking | 121 | 100 | 0.540 | 0.78(0.36-1.71) |
| Never drinking | 61 | 54 |  | 1 |
| Ever drinking | 108 | 90 | 0.543 | 1.15(0.73-1.81) |
| <58 | 85 | 67 |  | 1 |
| ≥58 | 84 | 77 | 0.954 | 0.98(0.53-1.82) |
| Female | 23 | 24 |  | 1 |
| Male | 146 | 120 | 0.84 | 1.06(0.60-1.86) |

**Table S14** The association between age, sex, drinking, smoking and rs2074356 in cancer free controls

|  | Controls(n=314) | | Adjusted *P* value | Adjusted OR(95%CI) |
| --- | --- | --- | --- | --- |
|  | CC | CT/TT |  |  |
| Never smoking | 116 | 42 |  | 1 |
| Ever smoking | 111 | 45 | 0.156 | 1.53(0.85-2.76) |
| Never drinking | 102 | 54 |  | 1 |
| Ever drinking | 125 | 33 | 0.004 | 0.43(0.24-0.76) |
| <58 | 122 | 45 |  | 1 |
| ≥58 | 105 | 42 | 0.853 | 0.95(0.57-1.60) |
| Female | 35 | 16 |  | 1 |
| Male | 192 | 71 | 0.919 | 0.96(0.45-2.08) |

**Table S15** The association between age, sex, drinking, smoking and rs2074356 in patients with ESCC

|  | Cases(n=313) | | Adjusted *P* value | Adjusted OR(95%CI) |
| --- | --- | --- | --- | --- |
|  | CC | CT/TT |  |  |
| Never smoking | 68 | 24 |  | 1 |
| Ever smoking | 128 | 93 | 0.196 | 1.54(0.80-2.95) |
| Never drinking | 78 | 37 |  | 1 |
| Ever drinking | 118 | 80 | 0.570 | 0.85(0.48-1.51) |
| <58 | 89 | 63 |  | 1 |
| ≥58 | 107 | 54 | 0.191 | 0.73(0.46-1.17) |
| Female | 39 | 8 |  | 1 |
| Male | 157 | 109 | 0.036 | 2.74(1.07-7.00) |

**Table S16** The association between age, sex, drinking, smoking and rs2274223 in cancer free controls

|  | Controls(n=314) | | Adjusted *P* value | Adjusted OR(95%CI) |
| --- | --- | --- | --- | --- |
|  | AA | GG/AG |  |  |
| Never smoking | 104 | 54 |  | 1 |
| Ever smoking | 105 | 51 | 0.767 | 0.92(0.54-1.57) |
| Never drinking | 99 | 57 |  | 1 |
| Ever drinking | 110 | 48 | 0.175 | 0.69(0.41-1.18) |
| <58 | 114 | 53 |  | 1 |
| ≥58 | 95 | 52 | 0.573 | 1.15(0.71-1.87) |
| Female | 36 | 15 |  | 1 |
| Male | 173 | 90 | 0.194 | 1.65(0.78-3.51) |

**Table S17** The association between age, sex, drinking, smoking and rs2274223 in patients with ESCC

|  | Cases(n=313) | | Adjusted *P* value | Adjusted OR(95%CI) |
| --- | --- | --- | --- | --- |
|  | AA | GG/AG | |  |
| Never smoking | 44 | 48 | | 1 |
| Ever smoking | 128 | 93 | 0.201 | 0.67(0.36-1.24) |
| Never drinking | 56 | 59 |  | 1 |
| Ever drinking | 116 | 82 | 0.163 | 0.67(0.38-1.18) |
| <58 | 81 | 71 |  | 1 |
| ≥58 | 91 | 70 | 0.438 | 0.84(0.53-1.32) |
| Female | 25 | 22 |  | 1 |
| Male | 147 | 119 | 0.273 | 1.56(0.71-3.44) |

**Table S18** Interactions between snps and age, sex, drinking and smoking on the risk of ESCC

| Interactions | Adjusted *P* value | Adjusted OR(95%CI) |
| --- | --- | --- |
| Rs738722*age | 0.009 | 1.73(1.15-2.60) |
| Rs738722*sex | 0.971 | 1.01(0.71-1.42) |
| Rs738722*drinking | 0.03 | 1.55(1.04-2.29) |
| Rs738722*smoking | 0.007 | 1.67(1.15-2.43) |
| Rs2074356*age | 0.143 | 1.41(0.89-2.23) |
| Rs2074356*sex | 0.014 | 1.59(1.10-2.30) |
| Rs2074356*drinking | <0.001 | 2.71(1.69-4.32) |
| Rs2074356*smoking | <0.001 | 2.72(1.79-4.14) |
| Rs2274223*age | 0.027 | 1.61(1.06-2.46) |
| Rs2274223*sex | 0.052 | 1.41(1.00-1.99) |
| Rs2274223*drinking | 0.002 | 1.97(1.29-3.01) |
| Rs2274223*smoking | <0.001 | 2.23(1.49-3.32) |

**Table S19** Multivariate analysis of age, sex, smoking, drinking, HPV sero status, SNPs and the risk of ESCC

|  | Cases(n=313) | | Controls(n=314) | | Adjusted *P* value | Adjusted OR(95%CI) |
| --- | --- | --- | --- | --- | --- | --- |
|  | No. | % | No. | % |  |  |
| Age (years) |  |  |  |  |  |  |
| <58 | 152 | (48.6) | 167 | (53.2) |  | 1 |
| ≥58 | 161 | (51.4) | 147 | (46.8) | 0.072 | 1.37(0.97-1.92) |
| Sex |  |  |  |  |  |  |
| Female | 47 | (15.0) | 51 | (16.2) |  | 1 |
| Male | 266 | (85.0) | 263 | (83.8) | 0.001 | 0.34(0.21-0.66) |
| Tobacco smoking |  |  |  |  |  |  |
| Never | 92 | (29.4) | 158 | (50.3) |  | 1 |
| Ever | 221 | (70.6) | 156 | (49.7) | <0.001 | 2.90(1.91-4.39) |
| Alcohol drinking |  |  |  |  |  |  |
| Never | 115 | (36.7) | 156 | (49.7) |  | 1 |
| Ever | 198 | (63.3) | 158 | (50.3) | 0.006 | 1.76(1.18-2.62) |
| HPV status |  |  |  |  |  |  |
| − | 143 | (45.7) | 178 | (56.7) |  | 1 |
| + | 170 | (54.3) | 136 | (43.3) | 0.001 | 1.74(1.25-2.44) |
| rs738722^α^ |  |  |  |  |  |  |
| CC (Ref.) | 169 | 54.0 | 187 | 59.6 |  | 1 |
| TT+CT | 144 | 46.0 | 127 | 40.4 | 0.228 | 1.23(0.88-1.72) |
| rs2074356^β^ |  |  |  |  |  |  |
| CC (Ref.) | 196 | 62.6 | 227 | 72.3 |  | 1 |
| TT+TC | 117 | 37.3 | 87 | 27.7 | 0.012 | 1.58(1.10-2.26) |
| rs2274223^γ^ |  |  |  |  |  |  |
| AA (Ref.) | 172 | 55.0 | 209 | 66.6 |  | 1 |
| GG+AG | 141 | 45.1 | 105 | 33.5 | 0.001 | 1.83(1.30-2.58) |
